# Health-Related Quality of Life in Patients with Lung Cancer Applying Integrative Oncology Concepts in a Certified Cancer Centre

**DOI:** 10.1155/2020/5917382

**Published:** 2020-05-10

**Authors:** Anja Thronicke, Phillipp von Trott, Matthias Kröz, Christian Grah, Burkhard Matthes, Friedemann Schad

**Affiliations:** ^1^Research Institute Havelhöhe, Hospital Havelhöhe, D-14089 Berlin, Germany; ^2^Interdisciplinary Oncology and Palliative Care, Hospital Havelhöhe, D-14089 Berlin, Germany; ^3^Institute for Social Medicine, Epidemiology and Health Economic, Charité–Universitätsmedizin Berlin, Corporate Member of Freie Universität Berlin, Humboldt-Universität zu Berlin, Berlin Institute of Health, D-10117 Berlin, Germany; ^4^Institute for Integrative Medicine, University of Witten/Herdecke, D-58313 Witten/Herdecke, Germany; ^5^Lung Cancer Center and Department of Pneumology, Hospital Havelhöhe, D-14089 Berlin, Germany

## Abstract

**Background:**

Pretreatment health-related quality of life (HRQOL) is associated with survival outcome in lung cancer patients. There is a lack of systematic research on pretreatment HRQOL in lung cancer patients who receive integrative oncology (IO). We evaluated patient-reported outcomes in these patients at time of diagnosis at a certified oncology and lung cancer centre.

**Methods:**

The present analysis is a prospective real-world data study. Clinical and demographic data were obtained from the accredited Network Oncology cancer registry. Pretreatment HRQOL was evaluated (international standardized questionnaires) for people with all-stage lung cancer at first diagnosis that received IO consisting of standard therapy and multimodal add-on complementary concepts. Univariate and adjusted multivariate regression analyses were performed with *R. Results*. Eighty seven patients with all-stage lung cancer were eligible for the questionnaire analysis (median age 68.0 years, IQR 59.0–74.4). Thirty percent of the total cohort reported financial difficulties. Self-reported pretreatment financial difficulty was associated with younger age (*p*=0.007), pretreatment pain (*p*=0.006), anxiety (*p*=0.04), and low mood (*p*=0.03). Pain (*p*=0.03) and young age (*p*=0.02) in the early- and late-stage lung cancer were associated with financial difficulties.

**Conclusion:**

We suggest physicians screen lung cancer patients at working age (broadly aged ≤65 years) and/or who report increased pain at the time of diagnosis as they might be at particular risk for emotional, physical, and financial problems. Our results emphasize to address emotional and physical needs before and during early treatment in lung cancer patients as suggested in integrative and supportive cancer concepts.

## 1. Introduction

Lung cancer is the most common cancer worldwide (1.83 million estimated new cases, 12.9% of all cancers worldwide) and the most common cause of death from cancer worldwide (one cancer death in five, 1.59 million deaths, 19.4% of the total) [1]. Importantly, in economically developed regions, lung cancer is the leading cause of cancer death among women (210,000 deaths) followed by breast cancer (198,000 deaths) [1]. According to the lung cancer alliance, only 17% of patients diagnosed with lung cancer survive for five years compared to 65% or 99% in colon or prostate cancer [2]. However, five-year lung cancer survival rates have increased by up to 5% in several countries [3], and this trend is continuing due to new targeted therapies and immune-oncological treatments [4].

Pretreatment health-related quality of life (HRQOL) and its related parameters predict survival in lung cancer patients [5, 6]. The rationale for this relationship may be that patients with a better initial state of general symptoms and psychosocial well-being before treatment are at an advantage of keeping this state and surviving longer [7]. Pretreatment HRQOL data seem to provide the most reliable information to establish prognostic criteria for the treatment of patients with cancer [8]. Furthermore, compared to follow-up HRQOL evaluations, they are easier to assess and show disease-specific rather than treatment-specific characteristics [8]. HRQOL has become increasingly important in the comprehensive care and support of lung cancer patients.

Integrative oncology (IO) is a patient-centered, evidence-based field of comprehesive oncological care utilizing lifestyle modifications, mind-body practices, and natural products alongside guideline-oriented standard oncological treatment [9]. The aim of IO is to engage patients and their families to actively participate in the process of prevention, treatment, and survivorship of cancer. In this way, IO promotes health and proactively addresses symptoms and adverse effects caused by cancer or its treatment [10]. Alongside newer treatment options, IO concepts have gained growing interest in lung cancer management [11] as they aim to reduce standard treatment-related side effects and improve HRQOL [12, 13].

Little systematic research has been published so far on pretreatment HRQOL in lung cancer patients being treated within IO concepts. The present prospective observational real-world study was performed to contribute filling this gap. To supply high-quality standards and to make the results internationally comparable [14], the European Organization for Research and Treatment of Cancer Core Quality of Life (EORTC QLQ-C30) [15] and Hospital Anxiety and Depression Scale, German Version (HADS-D) [16] questionnaires were evaluated in the present study. They were answered by patients being treated in a certified German Lung Cancer Centre (LCC), which is annually audited by the country's largest scientific-oncological medical expert association, the German Cancer Association [17]. The objective of the study was the evaluation of HRQOL at the first diagnosis before treatment in all-stage lung cancer patients at a certified LCC.

## 2. Materials and Methods

### 2.1. Study Design

We conducted a prospective observational study between March 2012 and August 2017. We included patients who were 18 years or older and who gave written consent, with a primary diagnosis of stage I–IV lung cancer at the certified LCC (Gemeinschaftskrankenhaus Havelhöhe, Berlin, Germany) with complete clinical and demographic data as well as complete pretreatment EORTC QLQ-C30 questionnaires (version 3.0) and HADS-D (2011). Patients who did not give consent and patients with missing data were excluded from the study. Information on complementary treatment options and patient's choice were prerequisites for the application of complementary concepts in addition to conventional oncology treatment.

### 2.2. Ethical Issue

The study has been approved by the ethical committee of the Berlin Medical Association (Berlin—Ethik-Kommission der Ärztekammer Berlin). The reference number is Eth-27/10.

### 2.3. Sample Size Determination

It was assumed that eight explanatory variables were required to yield good results for binary outcome prediction. According to Harrell et al. [18], a minimum of ten cases per variable would yield a stable model for logistic regression modelling, leading to a total sample size of at least 80 patients for adjusted multivariate regression analysis.

### 2.4. Data Collection

Demographic data as well as information on diagnosis, histology, surgery, and previous treatment regimen were obtained from the accredited clinical Network Oncology registry (for further methodology information on the NO registry please see [19]). Data included details of guideline-orientated conventional oncological care and add-on complementary concepts.

### 2.5. Endpoints

The primary outcome of the study was to investigate patient-reported pretreatment psychological, social, and/or physical HRQOL outcomes at the first diagnosis in patients that were treated in a certified German lung cancer centre within the context of multimodal IO concepts. The secondary outcome was the explorative evaluation of different patient-reported HRQOL outcomes and their association factors.

### 2.6. Statistical Analysis

The questionnaires and their subscales were evaluated according to their scoring manuals [20]. To quantify the strength of the relationship between patient-reported outcomes and demographic as well as treatment variables and to reduce the risk of bias, a multivariable linear regression analysis was performed. This model was applied for each of the 5 numerical functioning and 9 numerical symptom outcomes of the EORTC QLQ-C30 questionnaire and for both numerical subscales (anxiety and depression with 7 four-stage items, respectively) of the HADS questionnaire at V0 (baseline, diagnosis) as the response variable. Missing data were not part of the analysis as patients with missing data were excluded from the study. In order to yield reliable model results and to abstain from overfitting, we introduced a regression subset selection (*R* package “leaps,” version 3.0) including exhaustive search to select for a subset of reliable variables. The model with an indicated number of variables with the highest adjusted *R*^2^ was chosen as the best model. Continuous variables were described as median with interquartile range (IQR); categorical variables were summarized as frequencies and percentages. Data distributions were inspected graphically using box plots and histograms and were arithmetically examined for skewness. *p* values <0.05 were considered to be significant. All statistical analyses were performed using the software *R* (version 3.3.0, *R* Core Team).

## 3. Results

### 3.1. Patient Characteristics

Complete data (complete clinical and questionnaire data) were collected for 87 eligible patients with all-stage lung cancer receiving IO care. The response rate was 12.5% (87/698); reasons for nonparticipation were refusal to be included in the study due to a deteriorated general condition of the patient and incomplete data.

Table 1 shows the main characteristics of patients at baseline. Patients had an average age of 68.0 years, and the majority of the patients were diagnosed with advanced lung cancer (*n* = 44, 50.5%). Most of the patients were current or past smokers (*n* = 66, 75.9%), and adenocarcinoma was the histology in the majority of the patients (*n* = 47, 54%).

### 3.2. Oncological Conventional and Complementary Treatment

Concerning the conventional treatment (Table 2), the majority of the patients received surgery (*n* = 48, 55.2%) and systemic therapy (*n* = 51, 58.6%).

Almost half of the cohort received radiation (*n* = 41, 47.1%). With respect to nonpharmacological interventions, the greatest proportion of patients received psycho-oncological treatment (*n* = 54, 62.1%), followed by nursing (*n* = 45, 51.7%) as well as movement therapies and physiotherapy (*n* = 28, 32.2%, respectively). In addition to standard oncological systemic therapy, 41 patients (47.1%) received *Viscum album L*. (mistletoe) extracts.

### 3.3. Patient-Reported Outcomes

Baseline EORTC QLQ-C30 results for the total cohort (Table 3) were within the range of formerly published EORTC QLQ-C30 reference values for lung cancer patients [15]. Earlier analysis of our group revealed that financial burden at the first diagnosis was the only HRQOL variable that was significantly associated with the application of IO treatment (univariate: *β* = 23.3, *p*=0.001; multivariate: OR 13.9, 95% CI: 1.5–131.2, *p*=0.02), and that younger age was one of the main drivers for this association. We therefore concentrated on financial burden, as a pretreatment HRQOL outcome, and its associated factors during our further analysis. We found that 26 patients (29.9%) of the total cohort reported financial difficulties (Table 3).

### 3.4. Association between Financial Burden and Prognostic Factors

**Figure 1:**
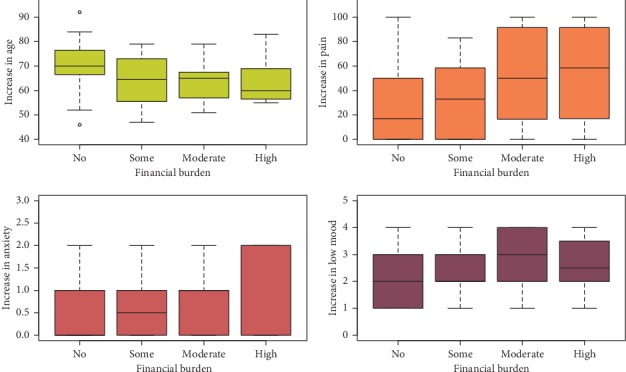
Interdependence (correlation) of pretreatment financial problems and young age and increased pain and stress, *n* = 87; (a) age (*β* = −1.0, *p*=0.007), (b) pain (*β* = 0.03, *p*=0.006), (c) anxiety (*β* = 8.6, *p*=0.05), and (d) low mood (*β* = 7.3, *p*=0.04).

We further explored factors that were associated with the variable, financial burden. We observed that young age (*β* = −1.0, *p*=0.007), pain (*β* = 0.03, *p*=0.006), anxiety (*β* = 8.6, *p*=0.0496), and low mood (*β* = 7.3, *p*=0.04) were significantly associated with the reporting of increased financial burden at the first diagnosis (Figure 1).

Advanced tumor stage at the first diagnosis did not correlate with increasing financial difficulties (UICC II vs. I *p*=0.11; UICC III vs. I *p*=0.9; UICC IV vs. I *p*=0.8). We examined financial distress in distinct tumor stage groups and how it correlated with factors such as age, pain, and emotional outcomes. We divided the patient's cohort into two subgroups—early tumor stage (UICC stage I-II) subgroup (*n* = 36) and advanced tumor stage (UICC stage III-IV) subgroup (*n* = 44).

### 3.5. Association between Financial Burden and Prognostic Factors according to Early Tumor Stage Groups

Table 4 shows univariate and adjusted multivariate linear regression results of the two subgroups, early and advanced tumor stage groups.

**Figure 2:**
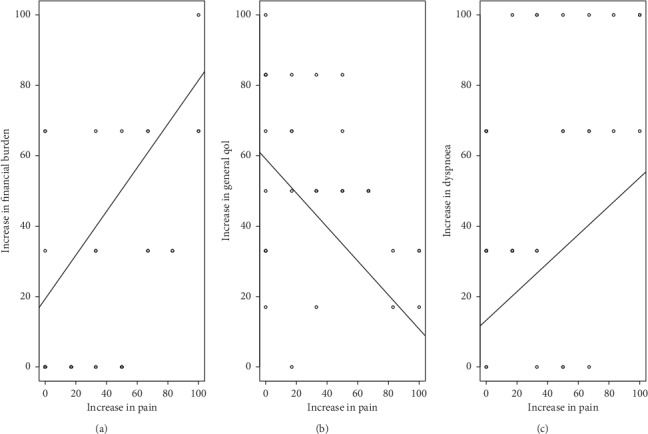
Correlation of pretreatment pain with (a) financial burden, (b) general quality of life, and (c) dyspnoea in UICC stage I-II patients, *n* = 36.

Univariate analysis revealed that significantly associated variables for the outcome financial burden in the early tumor stage subgroup were increased pain (*p*=0.0003), increased feeling of low mood (*p*=0.01), reduced global health (*p*=0.03) and general quality of life (*p*=0.04), increased insomnia (*p*=0.005), and depression (*p*=0.02). Pain (*p*=0.03) remained the only significant independent variable during adjusted multivariate analysis that was associated with financial difficulties.  Figure 2 shows the significant relationship between pain and increased financial difficulties (*p*=0.0003), reduced general quality of life (*p*=0.04), and increased dyspnoea (*p*=0.013) in the early tumor stage (stage I-II) subgroup.

### 3.6. Association between Financial Burden and Prognostic Factors in the Advanced Tumor Stage Groups

As shown in Table 4, for the advanced tumor stage subgroup (stage III-IV), univariate analysis revealed younger age (*p*=0.03), increased low mood (*p*=0.01), increased anxiety (*p*=0.049), and application of add-on *Viscum album* L. extracts (*p*=0.04) as significant associated variables for pretreatment financial burden of which only younger age (*p*=0.03) remained as an independent significant associated factor in adjusted multivariate analysis.

## 4. Discussion

Studies suggest that pretreatment HRQOL in lung cancer patients is predictive for survival. In this study, we evaluated pretreatment HRQOL in lung cancer patients receiving treatments within IO concepts. Thirty percent of lung cancer patients report an increased financial burden at diagnosis independent of their tumor stage. This burden is associated with patient's lower psychological and physical health. Our study is one of the first to examine the association between financial burden, age, and physical and emotional patient-reported outcomes in all-stage lung cancer patients applying IO therapies.

Most of the patients were diagnosed at an advanced stage of lung cancer. Patients facing a cancer diagnosis at this stage seek all possible treatment options including complementary therapies [21]. This is well reflected in our patient cohort as more than two-thirds of our lung cancer patients applied complementary concepts in addition to standard oncological therapy. This is in line with other studies on cancer patients receiving IO therapies [22–26]. Patients receiving IO in our certified lung cancer centre were not notably different to lung cancer patients treated in other German-certified lung cancer centres with respect to their baseline characteristics, self-reported HRQOL [15], and oncology treatment outcomes. In a former nationwide analysis comparing the performance status of our certified lung cancer centre to other benchmarking centres, also in terms of applied standard oncological therapies, we could show comparable and good results [27].

One-third of our patients reported financial difficulties at diagnosis. This is in line with the recent literature [28]. Financial burden was the strongest independent predictor for poor quality of life among cancer survivors, and the magnitude of this burden was a more significant HRQOL predictor than demography, education, ethnicity, and income of the family [29]. Financial problems in patients with lung cancer are negatively associated with HRQOL [30, 31] especially during early treatment, an observation that correlates with the results of our study.

Pretreatment financial difficulties in the present study were associated with increased anxiety and low mood, reflecting the results of a previous study of 1.278 US patients where financial problems correlated with low mood and anxiety [32]. A two-year prospective cohort study of 725 cancer patients found that financial difficulty was the main distinguishing characteristic between patients with persistent anxiety levels compared to all other anxiety groups [33]. The diagnosis of cancer can lead patients into an existential crisis with feelings of loss of security accompanied by fear and despair [34]. Lung cancer patients compared to thirteen other types of cancer were shown to experience the highest stress level [35].

In our study, increased financial difficulties were associated with pain in patients with early (stage I-II) lung cancer, and this association remained significant after adjusted multivariate analysis. Physical functioning which is determined by pain can among other variables be a significant predictor for survival of NSCLC patients [5]. A survey of 950 survivors with various cancer found that patients with cancer-related pain had significantly increased financial difficulties [36]. This had an impact on all EORTC function scales and three EORTC symptom scales including fatigue, sleeping disturbance, and appetite. Interestingly, the female gender was significantly associated with pain. Furthermore, a significant association between cancer and financial difficulties was observed in women reporting higher financial burden because they were less likely to be insured, more likely received cost-intense nursing outside home, and had more problems in paying for healthcare. Women also reported more pain, flares of pain, disabilities due to pain, and depression than men [36]. As lung cancer has recently emerged as the most common cause of death among women in economically developed areas, the cancer-associated symptom burden of female lung cancer patients needs to be acknowledged and treated. In contrast, in our study, the female gender was not associated with pretreatment financial difficulties. The healthcare systems in the US and Germany differ. Germany has the most restriction-free and consumer-oriented healthcare system in Europe, compensating for standard treatment care irrespective of patients' employment status [37]. Another explanation could be that while Germany has a higher percentage of women employed or partially employed than US, it is mostly men that contribute to the main income of the family. Therefore, the fear or burden of losing the main family income may be lower when a female family member has been diagnosed with a lung cancer diagnosis. Furthermore, differences between financial healthcare costs to be paid by the patient have to be taken into consideration when comparing financial burden of patients from various countries. The reimbursement of IO concepts may also differ from country to country. For example, IO concepts (including physiotherapy, nursing procedures, and psychological interventions) are mostly reimbursed by German statutory health insurance funds [38]. In Germany, the access to the healthcare system does not depend on the economic standing of the patient, and the out-of pocket costs for IO concepts are rather low. Thus, one has to differentiate between financial burden (in terms of anxiousness, as reported here) and real financial losses due to major financial cutbacks (in terms of, financial toxicity).

Our data reveal that increased financial difficulties were associated with younger age in stage III-IV lung cancer patients, and this association remained in the adjusted multivariate analysis. It has been evident that the age frame for “young” varies between cancer entities [27]. “Younger patients” for lung cancer in the present study meant that patients were ≤65 years representing the current working age threshold in Germany. Younger age as an associated factor for HRQOL has been associated both with better or worse health than older patients [25, 39–42]. In the present study, deterioration of financial difficulty was clearly associated with younger age as the diagnosis may result in loss of income as well as loss of important professional and family roles during that age. Employed or retired patients are reported to have higher HRQOL in physical and emotional dimensions compared to unemployed and disabled patients [42]. Further studies are required to investigate financial burden in unemployed and disabled patients.

Limitations of our study include the nonrandomized and cross-sectional nature of the design which is prone to confounding and selection bias. The external validity of the study is limited by the low response rate, and outcomes may be skewed by healthier lung cancer patients that perform better as they were able to answer the questionnaires. The confounding bias was reduced by adjusted multivariate regression analysis. The risk of bias due to missing data was reduced by excluding patients with missing data. In addition, our observation would need to be extended to longitudinal observations and a greater proportion of lung cancer patients in the future to make the results of the study more generalizable to lung cancer patients. Nevertheless, this study shows factors associated with HRQOL at diagnosis.

Pretreatment financial burden has been shown by recent studies to be the strongest independent predictor for poor HRQOL. We therefore suggest physicians to screen early (at the first diagnosis) especially young late-stage (III-IV) lung cancer patients who are of working age (broadly aged ≤65 years) and early-stage (I-II) patients with enhanced pain as they are at particular risk for emotional, physical, and financial problems. Early integration of supportive or palliative care with the adaptation to patient's specific needs improves HRQOL including the reduction of depressive symptoms in patients with metastatic lung cancer [43–45].

## 5. Conclusions

The results of the present prospective real-world study indicate an association between pretreatment financial burden and younger age in stage III-IV lung cancer patients and an association of increased financial burden with increased pain in stage I-II lung cancer patients. Pain as a consequence has an influence on the deterioration of a number of physical and emotional outcomes. Pretreatment financial burden may therefore serve as a predictor for increased pain and emotional burden and *vice versa*. Our results emphasize to address emotional and physical needs before and during early treatment in lung cancer patients including integrative and supportive cancer concepts.

## Figures and Tables

**Table 1:** Demographic data and characteristics at baseline.

	*n* (%)
Total number of patients	87
Age (years), median (IQR)	68.0 (59.0–74.4)
Gender, female	47 (54.0)

UICC stage	
I	13 (14.9)
II	23 (26.4)
III	17 (19.5)
IV	27 (31.0)
NA	7 (8.0)
BMI, mean, kg/cm^2^	25.0

Smoker	
current/past	66 (75.9)
Never	13 (14.9)
NA	7 (8.0)

Histology	
SCQ	19 (21.9)
ADC	47 (54.0)
LCC	2 (2.3)
NSCLC-NOS	2 (2.3)
Non-NSCLC	15 (17.2)

Demographic data and characteristics of lung cancer patients at baseline, *n* = 87.

**Table 2:** Oncological treatment and nonpharmacological interventions.

	*n* (%)
Total number of patients	87
Surgery	48 (55.2)
Systemic therapy^1^	51 (58.6)
Radiation	41 (47.1)
*Viscum album* L. extracts	41 (47.1)
Nonpharmacological interventions	70 (80.5)
Psycho-oncological treatment	54 (62.1)
Nursing (embrocation)	45 (51.7)
Movement (eurythmy therapy)	28 (32.2)
Physiotherapy	28 (32.2)
Massages	26 (29.9)
Music therapy	25 (28.7)
Breathing therapy	22 (25.3)
Drawing therapy	9 (10.3)

Pharmacological and nonpharmacological interventions of all-stage lung cancer patients (*n* = 87) ^1^Systemic therapy including chemotherapy, targeted therapy, and monoclonal antibody therapy.

**Table 3:** Summary of the questionnaire score at baseline.

		Mean	(SD)	(IQR)	(IQR)_ref_^1^
Global health status/QoL	QL	49.40	23.80	33.0–67.0	41.7–66.7
Physical functioning	PF	58.92	24.67	40.0–80.0	46.7–86.7
Role functioning	RF	48.48	32.53	17.0–67.0	33.3–83.3.
Emotional functioning	EF	54.80	27.34	33.0–75.0	50–83.3
Cognitive functioning	CF	74.08	24.05	67.0–100.0	66.7–100
Social functioning	SF	53.64	33.05	33.0–83.0	50–100
Fatigue	FA	56.46	29.48	33.0–78.0	22.2–66.7
Nausea and vomiting	NV	14.57	21.99	0–25.0	0–16.7
Pain	PA	36.59	33.69	0–58.5	0–66.7
Dyspnoea	DY	59.78	36.22	33.0–100.0	0–66.7
Insomnia	SL	43.72	35.22	0–67.0	0–66.7
Appetite loss	AP	40.97	37.44	0–67.0	0–66.7
Constipation	CO	27.94	36.43	0–50.0	0–33.3
Diarrhoea	DI	15.67	28.95	0–33.0	0–0
Financial difficulties	FI	29.10	33.89	0–50.0	0–33.3

Summary of the 15 scale scores of the EORTC QLQ-C30 questionnaire at baseline for all-stage lung cancer patients (*n* = 87) and comparison to EORTC QLQ-C30 reference values for lung cancer. ^1^Reference values provided by the EORTC Quality of Life Group Members and others of the QLQ-C30 [15]. QOL, quality of life; SD, standard deviation; IQR, interquartile range.

**Table 4:** Association factors for the outcome financial difficulties in stage I-II and in stage III-IV lung cancer patients.

	UICC stage I-II (*n* = 36)		UICC stage III-IV (*n* = 44)	
	Univariate *β* (S), *p* value	Multivariate *β* (S), *p* value	Univariate *β* (S), *p* value	Multivariate *β* (S), *p* value
Age	−1.0 (0.60), 0.09	0.1 (0.6), 0.8^1^	−1.1 (0.5), 0.03^*∗*^	−**1.2 (0.5), 0.02**^*∗*^^1^
Gender	−9.6 (10.26), 0.4	−4.9 (9.3), 0.6^1^	−9.9 (11.3), 0.4	−2.7 (11.2), 0.8^1^
Pain	0.5 (0.12), 0.0003^*∗∗*^	**0.5 (0.2), 0.03** ^*∗*^^1^	0.2 (0.15), 0.3	0.1 (0.2), 0.4^1^
Low mood	11.5 (4.3), 0.01^*∗*^	2.2 (5.5), 0.7^1^	11.5 (4.2), 0.01^*∗*^	6.0 (6.2), 0.3^1^
Global health	−0.5 (0.2), 0.03^*∗*^	−0.3 (0.2), 0.22^2^	0.2 (0.2), 0.3	0.4 (0.0.3), 0.13^3^
General QOL	−0.4 (0.2), 0.04^*∗*^	−0.2 (0.2), 0.32^2^	0.1 (0.2), 0.6	0.2 (0.3), 0.53^3^
Insomnia	0.4 (0.1), 0.005^*∗∗*^	0.2 (0.1), 0.22^2^	−0.0002 (0.2), 1.0	−0.1 (0.2), 0.63^3^
Depression	14.2 (5.8), 0.02^*∗*^	−2.4 (8.5), 0.82^2^	8.3 (6.2), 0.19	6.8 (6.0), 0.63^3^
Anxiety	10.9 (6.5), 0.1	4.8 (7.1), 0.52^2^	12.3 (6.1), 0.05	8.0 (6.9), 0.33^3^
Add-on VA	12.5 (11.7), 0.3	10.2 (10.6), 0.3^2^	24.3 (11.2), 0.04^*∗*^	23.0 (39.5), 0.2^3^
Dyspnoea	0.1 (0.15), 0.5	−0.2 (0.2), 0.3^2^	0.1 (0.15), 0.5	0.05 (0.2), 0.8^3^
Appetite loss	0.1 (0.14), 0.3	−0.01 (0.1), 0.9^2^	0.001 (0.15), 1.0	−0.02 (0.1), 0.9^3^
Stress	9.6 (5.3), 0.08	−1.5 (6.1), 0.8^2^	8.7 (6.4), 0.2	7.5 (7.1), 0.3^3^

Association factors for the outcome: financial difficulties, EORTC QLQ-C30 questionnaire, version 3.0. univariate and multivariate linear regression modelling, QOL, quality of life, *β* = estimate, and *S* = error; ^1^Adjusted for age, gender, pain, and low mood; ^2^Adjusted for age, pain, and low mood; ^3^Adjusted for age and low mood; add-on VA, additional *Viscum album* L. extracts; ^*∗*^*p* < 0.05; ^*∗∗*^*p* ≤ 0.005.

## Data Availability

The datasets used and/or analyzed during the current study have been kept confidential and are not available publicly. Additional data and materials may be obtained from the corresponding author on reasonable request.

## Abbreviations

EORTC QLQ-C30: European Organization for Research and Treatment of Cancer Core Quality of Life Questionnaire
HADS-D: Hospital Anxiety and Depression Scale, German Version
HRQOL: Health-related quality of life
IO: Integrative oncology
IQR: Interquartile range
NO: Network oncology
PRO: Patient-related outcome
UICC: Union for International Cancer Care
VA: *Viscum album* L.
